# Wastage from Diseases Due to Lice

**Published:** 1918-09

**Authors:** H. Ensor


**Wastage from Diseases Due to Lice.** Colonel H. Ensor, R. A. M. C.

The speaker said in part :

A very considerable source of preventable wastage from the divisional point of view is disease due to lice. In this group of sickness are (1) trench fever, (2) impetigo and I. C. T.

With regard to trench fever, this disease was of very great interest as it is the one disease which can fairly be said to be entirely new to medicine and to have been discovered as a result of this war.

The earliest work on it was, the speaker believed, done by Captain J. W. McNee, R.A M.C., who first transmitted trench fever to healthy men by the inoculation of the white blood from cases of this disease.

He also succeeded in reproducing the disease by the inoculation of washed red corpuscles, but failed with the blood plasma when hemolysis of the red corpuscles had occurred. Later the American bacteriologists succeeded in reproducing the disease by the inoculation into healthy men of plasma, urinary deposits, and the sputum from cases of trench fever. They also proved definitely that lice which had fed on cases of trench fever were the agents by which the disease was spread, and that it was the chief means of the transmission. They succeeded in reproducing trench fever in previously healthy men by means of rubbing the excreta and pounded up bodies of lice which had fed on cases of this disease, into scarified areas.

Captain J. F. Smith, R.A.M.C., has also recently called attention to the fact that the incidence of trench fever is much greater in men suffering from skin lesions due to pediculosis than in men suffering from skin diseases due to other causes.

The relation of lice to trench fever may be considered to be proved.

Impetigo and many cases of what is known in the British army as I.C.T. are also due to the irritation caused by lice and scabies. They can both be said to be diseases caused by the patient's scratching, thus bringing about a streptococcic infection of the skin. The speaker believed, at any rate, that at first the infection was streptococcic. Later the flora might be mixed.

The measures to be taken with regard to the prevention of these sources of wastage must obviously be those which exterminate lice among the troops of a division.

The extermination of lice under such conditions is far from easy. It is attempted in the field by means of bathing the troops and issuing to them clean underclothing, if possible, at least once in every ten days. With this object in view, baths are established in the divisional area, and laundries as near at hand as conditions permit.

Simple bathing of the men and the issuing of clean underclothing is, nevertheless, not enough. We know that lice habitually deposit their eggs on the body hair, particularly on the pubic and axillary hair. To destroy these eggs it is necessary to swab the parts after bathing with some solution calculated to do so. Hydrarg. perclor. 1/2 gr. dissolved in 1 oz. 2 o/o acetic acid will do this. All men after bathing should have their pubic and axillary hairs swabbed with this solution. No cases of irritation have occurred from its use so far as the speaker was aware. Shaving of the body hair is very strongly objected to by the men.

Even when this has been done, the men are far from lice-free. Lice deposit their eggs in the seams of the men's trousers, and in bad cases of pediculosis the speaker has seen them in the seams of the jackets. The sterilization of the men's service dress must then be carried out at the same time as they are bathed. This is the great difficulty from a divisional point of view. Each division of the British army has one Foden lorry disinfector attached to it, but as all the men's dirty underclothing has to be disinfected in the divisional area before it is sent to the laundry, the Foden lorry disinfector has more than enough to do to disinfect this underclothing, and it is not available for disinfecting the service dress of the men.

It has been attempted to destroy the lice and the eggs in the service dress by means of ironing the clothing, paying particular attention to the seams. Ironing, even when skilfully done, is not satisfactory. Hot irons will kill the lice, but it is very doubtful if as many as fifty per cent of the eggs are destroyed by such means.

At present the sterilization of the men's service dress by means of hot air is being largely carried out by divisions in the field. Captain Jacobs, R.A.M.C., devised an efficient, though perhaps a little complicated, hot air chamber for this purpose. The best and most simple one the speaker has ever seen is one devised by Captain Orr of the Canadian medical service. The living lice and their eggs are destroyed in about twenty minutes by exposure to a temperature of $60^{0}$ C. and Captain Orr's chamber is said to produce a temperature much higher than $60\,^{o}$ C. One of these chambers is installed at each divisional bath and, as the men are bathing, their service dress is sterilized in the hot air chamber.

Clothing sterilized by such means is dry and warm, and can be put on immediately.

The objection to this form of sterilization is that the apparatus is not mobile, and requires a considerable time to erect. In consequence, during an advance or a retirement, there are no means by which the service dress can be sterilized. This can best be done by attaching another Foden lorry disinfector for this special purpose to each division in the field. The disinfectors should be used for nothing else except the sterilization of the men's service dress and blankets as frequently as possible, although blankets are not particularly guilty of causing pediculosis.

This complaint is not a very serious cause of wastage in a good division, in which the men are previously inspected by their medical officers. If inspections are frequent and thoroughly carried out, cases are seen early and sent to a divisional or corps scabies station for treatment, at which place they can be cured in a few days and returned to duty. The treatment for scabies now considered the best is the old one of a daily hot bath with a thorough scrubbing with soft soap followed by inunction with a sulphur ointment. This done every day for three days in mild cases results in cure. If inspections are thoroughly carried out, there should be only mild cases to treat.

Each divisional or corps rest station is equipped with a steam disinfectant on the Servian barrel system, and there is no difficulty about disinfecting the clothing of scabies cases.

